# Subclinical hypothyroidism in children: is it always subclinical?

**DOI:** 10.1186/s13052-018-0462-4

**Published:** 2018-02-17

**Authors:** R. Gallizzi, C. Crisafulli, T. Aversa, G. Salzano, F. De Luca, M. Valenzise, G. Zirilli

**Affiliations:** UOC Pediatria, Department of Human Pathology of Adulthood and Childhood, University of Messina, via Consolare Valeria, 98125 Messina, Italy

## Abstract

Aim of this commentary is to report current knowledges on the main clinical and metabolic abnormalities which might be observed in children with longstanding and untreated subclinical hypothyroidism (SH) and to comment the most recent views about natural evolution of thyroid function in the cases with either idiopathic or Hashimoto’s thyroiditis-related SH. On the basis of these preliminary remarks, the essential guidelines for an appropriate and tailored management of SH children are also proposed.

## Background

Subclinical hypothyroidism (SH) is a condition that is also known as isolated hyperthyrotropinemia [1] and is characterized by serum TSH levels above the upper limit of the reference range, in presence of normal FT4 concentrations [2].

In adults SH is a relatively common disorder, which may be encountered in 4–20% of cases and shows a tendency to progress to overt hypothyroidism [3]. Moreover, it is frequently associated with important adverse effects, such as insulin resistance [4], dyslipidemia [5], diastolic and endothelial dysfunction [3, 6], coronary disease and heart failure [7–10]. Therefore, replacement therapy with L-T4 is frequently recommended for adult patients with either TSH serum concentrations > 10 m IU/l or TSH < 10 m IU/l and symptoms suggestive of thyroid failure [11, 12].

In children, on the contrary, SH seems to be less common, with a prevalence that has been reported to range around 1,7% [13]. Furthermore, several pediatric studies suggest that SH is a benign and remitting condition, with a negligible risk of progression to overt hypothyroidism [14] and controversial association with adverse health outcomes [15]. Therefore, the benefits of L-T4 treatment in children are clear only for the severe form of SH, whilst they are very uncertain for the mild forms [15]. However, although the available findings are insufficient to recommend replacement therapy for all the children with mild and asymptomatic SH, nevertheless current literature highlights the potential need for assessment of the subtle abnormalities, which may be associated with even modest increase in serum TSH levels [15].

This commentary aims to review current knowledges on the main clinical and metabolic abnormalities that might be observed in children with longstanding and untreated SH, in order to shed further light on the advantages of an individually tailored therapeutic management of the young patients with this disorder.

### Neurocognitive outcome

Thyroid hormones (TH) are known to play a pivotal role in the regulation of brain maturation and function. In fact, newborns and infants with untreated hypothyroidism are at risk of permanent mental retardation. Furthermore, even children aged more than three years at the time of hypothyroidism onset are at risk of developing subtle cognitive impairment, although TH-dependent brain development is already complete at that age.

According to the results of the few cross-sectional studies aiming to evaluate the influence of SH on cognitive function, the only subtle negative effects which may be observed in SH children concern attention level [16, 17], whereas cognitive performance has been reported to be absolutely normal in these children [13, 16, 17].

According to the results of the only prospective and case-control study on this topic, no differences seem to exist in verbal, performance and full-scale IQ scores, between SH children and a control group matched for socioeconomic status [18]. Moreover, no relationships were detected between IQ scores and either severity or duration of SH and no significant differences between SH children and controls were found, even in terms of psychologic and behavioural assessments [18].

Therefore, it is possible to infer, on the basis of the current literature on this issue, that SH is unable to negatively affect cognitive function, at least in childhood. This inference is also supported by the results of the small and short-term study by Aijaz et al. [16], according to which L-T4 treatment does not seem to be able to improve the attention problems that had been found in the children with untreated SH.

### Linear growth

TH are well assessed to play an important part in the regulation of growth and bone maturation; therefore, short stature and bone age retardation are recognized as two common manifestations of untreated overt hypothyroidism.

By contrast, no impairment in linear growth and bone maturation is generally reported in children with mild SH. In fact, height, growth velocity and bone maturation in a series of 36 children with longstanding SH were found to be normal and to not differ from those detected in an age-matched control population [18]. Furthermore, no changes in height velocity were documented in a large cohort of children with idiopathic and mild SH, who were followed for two years [19].

Finally, from the analysis of the current studies on the auxological repercussions of SH, it may be argued that a growth failure might be observed only among SH children with very severe thyroid dysfunction (TSH > 50 m IU/l and T4 levels at the low limit of the reference range), whilst no negative impact on growth velocity is detectable in children with mild SH [15].

### Bone health status

It is generally accepted that thyroid status, as well as genetic, ethnic, nutritional and lifestyle factors, is able to significantly affect bone mineral homeostasis,. Whereas hyperthyroidism increases bone turnover and the risk of osteoporosis [20], hypothyroidism reduces bone turnover, thus favouring a gain in bone mass and mineralization [21]. However, studies on large adult populations documented an increased risk of fractures in both the patients with overt hyperthyroidism and those with overt hypothyroidism [22, 23].

To the best of our knowledge, there is only one study aiming to investigate the bone mineral status in children with SH. On the basis of this study results, the authors concluded that bone health, as assessed both at lumbar spine and at phalanges of the hand, is not impaired in untreated children and adolescents with long lasting SH [24]. This inference has been subsequently highlighted by Salerno et al. in a recent review aiming to analyze the metabolic and clinical risk factors associated with SH in childhood [15].

### Obesity

It is well known that a biochemical picture of isolated hyperthyrotropinemia may be observed in around 10–23% of obese children [25], a relative prevalence which is distinctly higher than that generally reported in the pediatric general population, i.e. 1.7% [13].

The pathophysiological mechanisms which may be responsible for such a relationship between SH and obesity in pediatric age have not been clarified so far. However, it has been found that thyroid function may often normalize after weight loss [25]. Therefore, it is generally believed that SH is a consequence, rather than a cause, of weight gain [15].

It has to be emphasized, however, that the association between SH and obesity might play a key-role in conditioning an increased risk for metabolic syndrome. Indeed, both waist circumference and waist-to-height ratio were reported to be more elevated among obese children with mild SH than among their euthyroid counterparts and to correlate with TSH serum levels [26, 27].

### Cardiovascular function

SH in childhood has been reported to be associated with an increased risk of hypertension, whose prevalence was found to be significantly higher in SH children than in euthyroid controls [28, 29]. Nevertheless, these findings were not subsequently confirmed by other authors [26].

In adult patients SH was reported to be able to impair both flow-mediated dilation and intima-media thickness [6, 30]. By contrast, the only case control and prospective study about the effects of SH on vascular function in pediatric age failed to demonstrate any significant alterations in flow-mediated dilation and intima media thickness among 39 children with mild but long lasting idiopathic SH [31]. It has to be underlined that the children with SH, when compared with 39 euthyroid controls, exhibited, at entry, increased concentrations of asymmetric dimethylarginine, i.e. an aminoacid which may be considered as an early marker of endothelial dysfunction [31]. Serum levels of this aminoacid were subsequently found to normalize after 2 years of L-T4 therapy, thus suggesting that mild and untreated SH might be associated with early changes in pro-atherogenic profile [31]. In fact, although the SH children included in that study did not exhibit a biochemical picture of overt dyslipidemia, nevertheless the subtle alterations in HDL-cholesterol (C) and in triglycerides/HDL-C ratio, that were found at entry are incline to favour the onset of an atherosclerotic process. It is noteworthy that these alterations regressed under L-T4 treatment [31].

Similar proatherogenic abnormalities had been previously reported also by other authors, who investigated the relationships between SH and cardiovascular risk factors by cross-sectional study designs. Also the results of those studies suggested that TSH might be implicated in the regulation of both lipid profile and blood pressure, irrespectively of TH action [26, 32].

It might also be postulated that the proatherogenic abnormalities, which have been documented in SH children, could be ascribed to the presence of confounding factors, such as obesity and inflammation. Nevertheless, a multivariate analysis, aiming to identify the major determinants of the proatherogenic alterations found in SH children, revealed that the most independent factor associated with cardiovascular risk was the duration of SH [26]. On the basis of this finding, it was argued that longstanding untreated SH may be able to play a direct role on early atherosclerotic changes, not mediated by either visceral adiposity or other confounding factors [26].

### Natural course of SH

The most important factor, which is known to be able to affect the natural history of SH, is its etiology: either idiopathic or secondary to HT.

In the children with no underlying thyroid disorders natural evolution is characterized by a spontaneous normalization of thyroid function tests in a large majority of cases [33]. Therefore, idiopathic SH may be considered as a remitting or self-limiting process, with low risk of progression toward overt hypothyroidism [34, 35]. A deterioration of thyroid status over time might be predicted, at the time of SH diagnosis, in the cases with associated celiac disease and in those with initial presence of goiter and elevated thyroglobulin autoantibodies [36]. Baseline TSH levels, however, are probably the most powerful predictors of SH evolution over time [37, 38].

Whereas in children with idiopathic SH the risk of worsening their thyroid status over time is very low and the probability of a spontaneous TSH normalization is high, longitudinal course is very different in children with HT-related SH [33, 39, 40]. Furthermore, the children with HT-related SH have been found to be more exposed to the risk of developing a pathological enlargement of thyroid gland throughout follow-up [39], an adverse event which might be successfully counteracted with L-t4 therapy.

On overall, on the basis of the results of these prospective studies, it can be inferred that the association with HT is able to exert, per se, a negative impact on the long-term evolution of SH, irrespective of other concomitant risk factors [39]. Such a negative impact may be furtherly exacerbated by the coexistence of either Turner syndrome (TS) or Down’s syndrome (DS) [33], i.e. two relatively common chromosomopathies that are known to be associated with an increased risk of HT [41]. It is noteworthy that, in a significant percentage of DS children with HT-related SH, SH may progress over time to hyperthyroidism [33], a metamorphosis which may be observed, more rarely, even in children with no chromosomopathies [42].

### To treat or not to treat?

The question of whether individuals with SH should be treated or not is still controversial, owing to the lack of case-control studies documenting significant benefits of L-T4 treatment on hypothyroid symptoms, life quality, cardiovascular function and metabolic abnormalities [43]. It has to be clarified, however, that the decision of what to do regarding these patients should be based, in the first place, on patients’ age. In fact, a therapeutical policy is more justified, on the whole, for the adult patients with SH, who are more incline to deteriorate their thyroid status over time. By contrast, the risk of progressing from SH to overt hypothyroidism is distinctly lower in children and adolescents. Furthermore, cardiovascular and metabolic complications have been reported to have a more severe clinical expression in adults than in children [6, 30, 31].

Other aspects which have to be evaluated, when an endocrinologist considers the opportunity of starting L-T4 therapy in SH patients, concern the severity of thyroid dysfunction. In many pediatric centres, in fact, treatment is initiated for persistent elevations > 10 mIU/L or for progressively increasing TSH serum levels [44].

Finally, it has to be considered whether SH is idiopathic or HT-related. In this last event, in fact, the risk of a thyroid status deterioration over time is distinctly higher, especially in the children with a concomitant DS or TS and therefore a close monitoring of thyroid function is mandatory. On the contrary, in the children with neither underlying thyroid disorders, nor associated autoimmune diseases, nor chromosomopathies, the risk of a thyroid status worsening is not very high and the hypothesis of a treatment should be taken into consideration only in the cases with hypothyroid clinical symptoms, or thyromegaly, or very elevated TSH serum levels at entry [36]. In fact, in the patients with idiopathic SH, the evidence for a benefit of L-T4 supplementation is very limited, at least in pediatric age [44]. The only study comparing the effects of treatment versus no therapy in children with idiopathic SH seems to suggest that L-T4 therapy is unable to modify post-treatment outcomes of hyperthyrotropinemia and to prevent the risk of a further TSH increase after treatment withdrawal [38].

## Conclusions

SH in children and adolescents may not be always asymptomatic. In a limited number of cases with longstanding SH, cardiovascular abnormalities and proatherogenic metabolic alterations may be sporadically observed, especially in the patients with associated obesity. The risk of such complications should be considered when a decision about the management of SH has to be taken. It is also important to consider whether SH is idiopathic or HT-related. Furthermore, it should be considered the possible association with other factors, that are known to exert a negative impact on the long-term evolution of this disorder.

## Abbreviations

C: Cholesterol
DS: Down’s syndrome
HT: Hashimoto’s thyroiditis
L-T4: Levo-thyroxine
SH: Subclinical hypothyroidism
TS: Turner syndrome
